# Adolescents and young adults’ (AYA) views on their cancer knowledge prior to diagnosis: Findings from a qualitative study involving AYA receiving cancer care

**DOI:** 10.1111/hex.13170

**Published:** 2020-12-04

**Authors:** Ruth I. Hart, Fiona J. Cowie, Angela B. Jesudason, Julia Lawton

**Affiliations:** ^1^ Usher Institute, Medical School University of Edinburgh Edinburgh UK; ^2^ Beatson West of Scotland Cancer Centre Glasgow UK; ^3^ Department of Paediatric Haematology and Oncology Royal Hospital for Sick Children Edinburgh UK

**Keywords:** adolescents, cancer, information, knowledge, patient care, qualitative research, young adults

## Abstract

**Background:**

Cancer is rare amongst adolescents and young adults (AYA). Previous research has reported (healthy) AYA’s knowledge of risk factors and symptoms as limited, with this potentially leading to delays in help‐seeking and diagnosis.

**Objectives:**

We explored AYA’s views on their cancer knowledge prior to diagnosis and if/how they perceived this as having affected their experiences of diagnosis and care.

**Methods:**

We interviewed 18 AYA diagnosed with cancer (aged 16‐24 years). Interviews were recorded and transcribed verbatim. We undertook qualitative descriptive analysis, exploring both a priori topics and emergent themes, including cancer knowledge prior to diagnosis.

**Results:**

Adolescents and young adults characterized their knowledge of cancer and treatment prior to diagnosis and treatment initiation as limited and superficial. AYA perceived gaps in their knowledge as having profound consequences throughout their cancer journey. These included: hindering recognition of symptoms, thereby delaying help‐seeking; impeding understanding of the significance of tests and referrals; amplifying uncertainty on diagnosis; and affording poor preparation for the harsh realities of treatment.

**Conclusions:**

Adolescents and young adults perceived their limited cancer knowledge prior to diagnosis as affecting experiences of diagnosis and initial/front‐line care. These findings prompt consideration of whether, when and how, AYA’s knowledge of cancer might be improved. Two broad approaches are discussed: universal education on AYA cancer and/or health; and targeted education (enhanced information and counselling) at and after diagnosis.

**Patient or Public Contribution:**

Our work was informed throughout by discussions with an advisory group, whose membership included AYA treated for cancer.

## 1 INTRODUCTION

Cancer is rare amongst adolescents and young adults (AYA), but its incidence is increasing.^1^ Its impact on young lives can be profound. A growing body of quantitative and qualitative work has considered AYA’s experiences of cancer, diagnosis and treatment/care.^2^, ^3^, ^4^, ^5^, ^6^, ^7^, ^8^, ^9^, ^10^, ^11^, ^12^, ^13^ That work suggests the experiences and needs of this age group differ both from those of older adults, who comprise the majority of cancer patients/service users, and of younger children.

We recently completed a study which explored AYA’s experiences of decision‐making about front‐line cancer treatment and their perspectives on participation in (Phase 3) clinical trials. That study highlighted significant challenges to informed decision‐making about both treatment and trials, including difficulties engaging with (disease and treatment‐related) information in the immediate aftermath of diagnosis.^14^ The early stages of our analysis revealed an additional, important theme: knowledge of cancer and its treatment *prior* to diagnosis. Identification of this theme prompted further, more detailed analytical work, which we report in this paper.

Previous research on AYA’s cancer knowledge provides a context for this analysis. That research has shown that (healthy) AYA have limited knowledge of key cancer risk factors and symptoms.^15^, ^16^, ^17^, ^18^ Studies have also highlighted deficiencies in (healthy) AYA’s knowledge of cancer‐related personal health surveillance (self‐examination) practices and public health initiatives (such as HPV vaccination and cervical screening programmes).^19^, ^20^, ^21^, ^22^, ^23^, ^24^, ^25^ It has been suggested that these knowledge gaps may lead to delays in symptom recognition, help‐seeking and diagnosis. Late diagnosis is commonly associated with poorer outcomes, and preventable delays are a prominent concern in the AYA cancer literature.^2^, ^5^, ^26^

However, prior research has focussed on understanding (typically measuring) *healthy* AYA’s knowledge of cancer. Very limited attention has been given to the views of AYA diagnosed with cancer about their pre‐diagnostic cancer knowledge and, whether and, how, they perceive this as having influenced their experiences of diagnosis and care. Understanding knowledge is important, as studies exploring the information (and other) needs of AYA with cancer further from diagnosis and/or treatment suggest that misconceptions and gaps in knowledge about cancer and its treatment can have costs for AYA later in life. Several authors report associations between unmet information needs and psychological distress or poor Health‐Related Quality of Life.^27^, ^28^, ^29^, ^30^, ^31^

Recent years have seen significant developments in AYA cancer care, and, in the United Kingdom (UK), AYA’s access to specialist (AYA) cancer services has increased.^32^ Nevertheless, scope remains to enhance AYA’s preparation for, and support them through, the substantial challenges presented by cancer diagnosis and treatment. Improved understanding of AYA’s prior knowledge (of cancer and treatment) might facilitate this, providing a foundation for the development of additional educational/informational interventions. Hence, in undertaking the analysis reported here, our objectives were to explore:

- AYA’s views on their cancer knowledge prior to diagnosis, including its nature, extent, and source(s);
- AYA’s views on whether and how this knowledge affected their experiences of diagnosis and care.

## 2 METHODS

In reporting our methodology/methods, we take direction from the Consolidated Criteria for Reporting Qualitative Studies (COREQ).^33^

### 2.1 Study design

Our work is underpinned by a critical realist perspective—a philosophical orientation combining a realist ontology and constructivist epistemology.^34^ Qualitative description, focussed on the identification and description of minimally‐theorized themes, provided a methodological framework for the study.^35^, ^36^ This involved an emergent, inductive design, purposive sampling, semi‐structured interviews and an iterative relationship between data collection and analysis. Work was informed throughout by discussions with study advisory group members, who included AYA treated for cancer. The South East Scotland Research Ethics Committee 01 approved the study (REC reference 17/SS/0077).

### 2.2 Study context

We undertook this study in Scotland, UK. Here, people diagnosed with cancer aged 16 or above typically receive treatment/care from oncologists and/or haematologists in a National Health Service (NHS) Scotland adult hospital. Some patients aged 16‐19 receive care from paediatric oncologists in a paediatric hospital. Dedicated AYA cancer/chemotherapy units, with specialist nurses and facilities, have been established (in adult and/or paediatric hospitals) in the four largest Scottish health boards. AYA living in other regions/health boards may be—and increasingly are—referred to these units/centres for treatment.

### 2.3 Study sample

Our approach to sampling was purposive, seeking variation in characteristics potentially relevant to participants’ experience(s) and perspective(s). These characteristics included age, gender, diagnosis and place of care. Our sample included AYA who were receiving/received front‐line care in all the types of care setting described above. More information on the sample is provided in Table 1.

**Table 1:** Participant characteristics

AYA with cancer (n = 18)	
Age: median (range)	
At diagnosis	19 (16‐24) y
At interview	20 (17‐26) y
Gender, male: n (%)	14 (78)
Ethnicity: n (%)	
White British	14 (78)
Non‐white British	2 (11)
White non‐British	2 (11)
Education/employment at diagnosis: n (%)	
Employment/work‐based training	6 (33)
School/college	6 (33)
Undergraduate studies	3 (17)
Not in education/ employment	3 (17)
Diagnosis: n (%)^a^	
Bone sarcoma	6 (33)
Leukaemia	4 (22)
Germ cell tumour	3 (17)
Other sarcoma	2 (11)
Lymphoma	1 (6)
CNS tumour	1 (6)
Melanoma	1 (6)
Diagnostic type: n (%)	
Primary cancer	16 (89)
Relapsed cancer	2 (11)
Place of care: n (%)^a^	
AYA cancer unit in an adult hospital	14 (78)
AYA cancer unit in a paediatric hospital	3 (17)
Adult cancer service in an adult hospital with no AYA cancer unit	1 (6)
Reported enrolment in a trial: n (%)	5 (28)
Interviewed without caregiver present: n (%)	14 (78)
Time from diagnosis to interview: median (range)	10 (2‐59) mo

Abbreviation: AYA, adolescents and young adults.

^a^ Percentages do not sum to 100% due to rounding.

### 2.4 Recruitment

Direct care team members made the initial approaches to AYA. These professionals outlined the study verbally and gave interested individuals a pack containing a participant information sheet, consent form and an opt‐in form with a pre‐paid envelope addressed to the research team. AYA interested in taking part were also given the option of contacting the project researcher (RH) by email or phone. RH followed up these expressions of interest, using contact details provided by AYA. This approach was intended to minimize pressure on AYA to participate, and the burden/work falling on direct care teams. However, it has meant we are unable to say how many potential participants were approached, or what their reasons for declining involvement were. Recruitment continued until our sampling ambitions were satisfied, and new topics and themes no longer emerged in new data.

### 2.5 Data collection

Interviews were conducted between November 2017 and December 2018, at a time and place chosen by AYA. This was typically their home or a private space at their usual treatment centre. Whilst AYA were usually interviewed on their own, interruptions (by family and/or health professionals) were common. Four chose to be interviewed with a caregiver present. Interviews were conducted by RH, a social scientist with 15 years’ experience of doing qualitative research, substantially relating to AYA’s experiences of health and illness. RH was not known to interviewees prior to the study but outlined her professional background and research interests, as well as checking AYA’s understanding of the project, before obtaining their written consent to participation. In the UK, people aged 16 years and above are entitled to consent to health research (and indeed to their own health care/treatment); hence, parental consent was not required. Interviews followed a topic guide, informed by literature reviews, and inputs from clinical co‐investigators and AYA advisors. A copy of the full topic guide is appended to our previous publication.^14^ Topics/questions of most relevance to this analysis were as follows: *Before your own diagnosis, what did you know about cancer and its treatment? Is there anything you wish you had known about cancer and its treatment? What have you learnt, since diagnosis, about cancer and its treatment?* The guide was revised over the course of the study to take account, and enable further exploration of, emerging themes. It was also adapted, in situ, in response to interviewees’ accounts and to give AYA scope to talk freely about issues they viewed as important. Typically interviews lasted one to two hours; all were recorded, with participants’ consent. Contemporaneous notes were taken, which included information on the environment/setting, interruptions and non‐verbal communication.

### 2.6 Data processing and analysis

**Figure 1:**
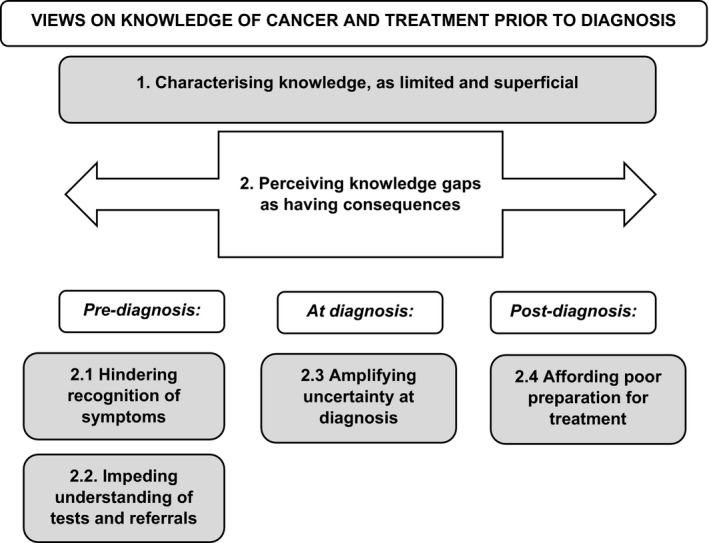
Emergent themes

Interviews were transcribed verbatim, but anonymized (i.e. participant identifiers were removed). Transcripts were then imported into the qualitative data‐indexing software NVivo (Version 11, QSR International Pty Ltd., Doncaster, Victoria, Australia). After familiarization with the transcripts (involving close reading and line‐by‐line coding), more focussed coding and analysis was undertaken by two members of the qualitative research team (RH and JL). Using a constant comparative approach,^37^ these individuals coded for topics, issues and themes. Mapping and memo‐ing strategies were used to define the content, parameters and relationships between codes. RH and JL prepared analytical reports, with these providing a basis for discussions to refine and agree coding and reporting frameworks. Whilst some topics were of a priori interest, and informed lines of questioning in all interviews, the themes/sub‐themes reported in this paper were largely emergent, that is derived from the data. These are outlined in Figure 1 and detailed in the Results section. Members of the wider research team confirmed that the identified themes were reflective of the data. Whilst interviewees were not asked to comment on individual transcripts, they, and other AYA diagnosed with cancer, were invited to provide feedback on key study findings via a workshop organized in the final stages of the project.

## 3 RESULTS

### 3.1 Study participants

We interviewed 18 AYA diagnosed with cancer whilst aged 16‐24 years and receiving care (active treatment and/or follow‐up) through an NHS Scotland oncology/haematology service. 17/18 were being (or had been) treated in a specialist AYA cancer/chemotherapy unit. Further information is provided in Table 1.

### 3.2 Study themes

Under the over‐arching theme of ‘(Views on) knowledge of cancer and its treatment prior to diagnosis’, we identified a series of contributory themes. These themes and their relationships are mapped out in Figure 1: those highlighted/shaded are then detailed in narrative form, below. We begin by documenting (Theme 1) how AYA characterized their knowledge of cancer and treatment ahead of their own diagnosis and care. We note where AYA identified important differences between their prior understanding and subsequent realities and consider their explanations for perceived gaps in knowledge. We then document (Theme 2) the consequences AYA perceived these knowledge gaps as having had: pre‐diagnosis (sub‐themes 2.1 and 2.2); at diagnosis (sub‐theme 2.3) and post‐diagnosis/on treatment initiation (sub‐theme 2.4). Theme headings are reflective of the content of participants’ accounts: verbatim quotes are indicated by inverted commas.

#### 3.2.1 Theme 1: Characterizing prior knowledge as limited and superficial

AYA consistently characterized their prior knowledge of cancer and its treatment as limited, superficial and/or abstract. Even those who portrayed themselves as having had ‘some’ previous knowledge typically drew attention to its shortcomings, using words such as ‘basic’, as illustrated by A04, a young man diagnosed shortly after starting university:

I knew the basics… I knew it was like, a serious disease, that affects your body's cells, like and the way that they divide and stuff. (A04)

Others described how it had only become clear to them retrospectively quite how deficient their knowledge had been. For example, one young man, interviewed towards the end of treatment, commented:

It's amazing like… if you think you know anything about cancer, or that, when you actually get it… what you think you know, is, like, a fraction of what is actually there… you just sort of see on the surface, for what it is, like this really negative, horrible thing. (A16)

Reflecting on their knowledge of cancer treatment, interviewees noted that this had been skewed towards chemotherapy and even here had been limited. For example, they had not appreciated the variety of regimens in use:

If you're not familiar with like cancers, then you just think chemotherapy's one thing. And that there's not like, hundreds of different types of chemotherapy… and that you could get them in tablet form and things like that. (A06)

Adolescents and young adults also reported limited prior knowledge of how chemotherapy (and other cancer treatments) worked, and what the potential long‐term implications of such treatments might be. Several, for instance, commented that they had been entirely unaware of the potential impact on their fertility:

(Going) back to like my, my lack of understanding about cancer and stuff, I had no idea fertility was an issue. (A15)

Discussion of the sources of AYA’s knowledge provided some explanation for its character and limitations. For example, interviewees recalled receiving some education about cancer and its treatment at school, which they now viewed as extremely rudimentary:

At primary school I got a book which was (called)… the “Big Book of Science” or something… and it had this, just this little bit about chemotherapy, which I found fascinating, and… this little like… pictogram of the coloured, drugs, finding the cells, and killing them… (A14)

In addition, AYA described learning about how cancer manifests, is experienced, and treated, via the media. Reflecting on how media portrayals had shaped their understanding and expectations of cancer treatment, AYA noted a focus on chemotherapy, and recalled TV and film depictions of people ‘hooked up’ to a machine, like ‘the guy in “Breaking Bad”’ (A14). In retrospect, AYA judged such portrayals as misleading:

You see stuff on the telly, like, and you think to yourself, “No, that's, that's not at all what it's like!” (A03)

TV (shows) cancer patients in bed, with a towel around their head… It's not what the TV, the films, make it out to be. (A11)

Typically, AYA talked of having had very little direct contact with other people with cancer prior to their own diagnosis. Some identified family members or family friends who had (had) cancer, but these were usually older, and often distant, relations with whom they had had little or no contact over the course of treatment:

(Girlfriend), her Gran unfortunately passed away with cancer a couple of years ago… that's the only time I’ve really experienced cancer. (A16)

Even when AYA reported knowing (of) another AYA with cancer, they seldom portrayed themselves as having accumulated and drawn on knowledge about that person's experience. Accounting for this—and their lack of knowledge more generally—interviewees explained that, until their own diagnosis, cancer had seemed a topic of little personal relevance:

You just never think it's gonna happen to you. Like you just think it's… one of these things, like, “Oh it's such a shame that (so‐and‐so) got diagnosed, but it's, no, it'll never happen to me!” (A05)

#### 3.2.2 Theme 2: Perceiving knowledge gaps as having consequences

### 3.3 Sub‐theme 2.1: Hindering recognition of (signs or) symptoms

Interviewees suggested that their limited knowledge had had consequences at different points in their cancer journey. These included not recognizing emerging signs/symptoms as potentially indicative of cancer. In particular, many interviewees reported not having appreciated the significance of non‐specific symptoms such as fatigue, which some described discounting for long periods:

Before like getting ill and that, I’d come home from school, and go for like a five‐hour nap. And… Mum'd be like, “Why are you going to bed?” And I’m like, “‘Cause I’m tired”. You never really think about things… you kind of just brush it off… you don't think (it might be cancer). (A18)

Others noted how they had remarked and reflected upon such symptoms, but—initially at least—attributed them to life pressures and/or lifestyle. For example, a young man diagnosed shortly after graduation described how he had ascribed fatigue to the demands of his examinations. Similarly, a young man who had been working in a bar when his health had started to deteriorate explained how he and a friend had attributed his cough to a hedonistic lifestyle:

I had this cough for quite some time… I told my friend… and she told me, “Just drink less”… (That) helped, so I thought, “Aah, it's not that bad. (Laughs) It can't be cancer, right?” (A13)

Adolescents and young adults described how these mundane explanations had encouraged stoicism, self‐management (eg rest, reduction in alcohol consumption) and monitoring. Typically, they reported seeking help from health professionals (eg visiting a GP) only once self‐care strategies had proved ineffective, symptoms became more pronounced and/or a caregiver intervened. By this point, some AYA said they had been very unwell. Even so, some noted how they had only given partial (selective) reports, prioritizing their most troubling and/or specific symptoms:

So I went to this doctor's appointment. Talked to the doctor, told her my symptoms. Now I wasn't, I didn't mention some of the things I’ve mentioned to you, because, I thought maybe they weren't that important, so, I think it would've definitely been hard for the doctor I saw to make, a complete diagnosis, just on my presenting complaints. However, she probably could've done a better job at fishing the information out of me, and maybe asking more questions. (A12)

### 3.4 Sub‐theme 2.2: Impeding understanding of the significance of referrals and tests

Adolescents and young adults suggested that their lack of knowledge had had consequences even after help from professionals had been sought. Whilst they often described referral to secondary care for specialist attention and/or further investigations as prompting a suspicion that something quite serious was wrong, few reported having considered cancer as a possibility until this was explicitly suggested by a health professional. Hence, even when scans/imaging, or more invasive investigations such as biopsies had been scheduled, this had not necessarily set off ‘alarm bells’, as the following young man, who had been experiencing pain and swelling in a lower limb described:

It's strange though, even though I had a biopsy, the idea that I might have cancer… barely crossed my mind. (A15)

Furthermore, AYA noted how they had not necessarily understood the results of investigations as being suggestive of cancer. Some remarked that the language professionals used to report the discovery of abnormalities had been unfamiliar to them. For example, A17, a young man ‘blue‐lighted’ from his local hospital to a larger regional facility for investigation of a suspected neurological cancer, explained that the term ‘mass’ had not held a clear meaning for him:

They said, “There's a mass in your brain,” or something. [Interviewer: And what did you take that to mean?] No idea… “A mass” – that could mean anything. (A17)

Adolescents and young adults viewed their limited appreciation of the significance of referrals, tests and results, as having important practical and psychological sequelae. For example, some reported that misunderstanding of the reasons for referrals and/or tests had led to them attending pivotal consultations alone, including those at which their cancer diagnosis was first disclosed:

I went by myself (to get the results of biopsy), because, like I say, I wasn't expecting… [Interviewer: That sort of news?] Yeah, I was, I thought it would be relatively benign. (A15)

Other AYA, ultimately diagnosed with haematological malignancies, described their bewilderment and/or distress on seeing ‘Cancer’ signs when admitted to hospital:

I was a bit confused. I didn't really know what was up with me. And then obviously at the (Cancer Centre), it says (Regional) Cancer Centre, whatever it is, underneath the (Hospital Name). So I was like, “Right, what's, what's going on?”. (A07)

### 3.5 Sub‐theme 2.3: Amplifying uncertainty at diagnosis

Adolescents and young adults explained how lack of awareness that cancer was a plausible explanation for their symptoms had left them both practically and emotionally ill‐prepared for diagnosis. Many suggested that the confusion, shock and distress of diagnosis had been compounded by their lack of knowledge of the types of cancer involved. Few AYA reported having had any familiarity with their/common AYA cancers (sarcomas, leukaemias, brain tumours, testicular and skin cancers). Some noted how, on receiving their diagnosis, they had not realized they were being told they had cancer:

When (Consultant Haematologist) told me first, It's, it's Hodgkin's Lymphoma, he put it, the name through Google Translator – and I still didn't know what it, what it was. (A13)

The word sarcoma isn't part of my vernacular so much that I instinctively identified it with cancer, so I needed a bit… of explaining, and then (the surgeon) said, “Your cancer is…” and I’m like, “Oh crap, right, so this is cancer?” (A15)

With a few exceptions (typically older interviewees), AYA portrayed themselves as having been profoundly unsure of the implications of their diagnosis, and the sort of future they might expect. Reflecting on the moment she learnt she had cancer, A18 explained:

(Mum) told me. She was like, “You've got cancer”. And I just… kind of sat there… It's just a… a word that you don't really know how to be… ‘cause, you know, you don't really know the outcome to that. (A18)

Nevertheless, AYA reported having had to rapidly make important decisions about their care and the practical organization of their lives.

### 3.6 Sub‐theme 2.4: Affording poor preparation for the realities of treatment

As reported in Theme 1, AYA perceived their prior knowledge of cancer treatment as having been minimal. AYA suggested that this too had had consequences, highlighting the gulf between their expectations of treatment and actual experience. For many, treatment had involved not only chemotherapy, but also radiotherapy and/or surgery. Several commented that they had been unaware of the duration of regimens (commonly months and for some AYA‐relevant cancers as much as three years). This, and/or the need for frequent and numerous visits including (for some) extended in‐patient stays, had been unexpected and dismaying:

The biggest shock… was the length of time that I was gonna have chemo… the fact that it was gonna go on for months and months was a bit of a surprise. (A08)

Though AYA acknowledged health professionals’ efforts to make them aware of the treatment plan and potential side effects, many said they had not grasped the pervasive and brutal impact treatment would have on their lives. With a few exceptions, AYA described treatment as far more debilitating and disruptive than they had anticipated. Several reflected on how, at diagnosis, they had viewed cancer as a ‘blip’ and treatment as a temporary disruption. Some described planning things to do during treatment, and, as A15 explained, ‘almost treating this like a sabbatical’. These AYA said they had soon realized that few of their ambitions were achievable:

As a symbol of how naïve I was, when I first got diagnosed… I was thinking about how I could do push‐ups, and little workouts in my room, to keep my body fit, and, I did that for like a week, and then I couldn't do it anymore. Because I had this vision of having my body intact, over this period, but, there was just, there was no chance of that happening. (A12)

In contrast to their initial expectations, AYA reported how their treatment regimens had proved all‐consuming. Some explained how the effects of treatment had presented challenges to the most fundamental aspects of daily life, such as taking a shower:

You don't realise the implications… all the ways that it affects you… like some of them are… they're so, I wouldn't say basic, but they're things that maybe we take for granted, on a daily basis kind of thing. (A16)

To better prepare AYA for the realities of treatment, some interviewees suggested that health professionals should have much franker conversations at the time a plan was discussed and agreed. However, others expressed different—and sometimes conflicting—attitudes to such information. These AYA surmised that knowing what lay ahead might have compounded their anxieties.

## 4 DISCUSSION

Our objectives in this analysis were to explore AYA’s views on their cancer knowledge prior to diagnosis, including if, and how, they felt that this knowledge had affected their experiences of diagnosis and care. In so doing, it was not our intention to suggest that knowledge is the only factor affecting experience, but simply a factor which, to date, has not perhaps received the attention it deserves. Our analysis revealed that AYA consistently characterized their knowledge as limited and superficial prior to diagnosis. We have further highlighted how AYA perceived inadequate knowledge as having had consequences prior to, at, and following diagnosis: hindering recognition of symptoms; impeding understanding of tests and referrals; amplifying uncertainty at diagnosis; and affording poor preparation for the harsh realities of treatment.

Whilst our research design does not equip us to confirm causal relationships, these findings have sensitized us to the potentially serious implications of (lack of) knowledge and prompted consideration of the possibilities for intervention. Three findings in particular warrant discussion: lack of knowledge may hinder symptom recognition, delaying help‐seeking and diagnosis (see sub‐theme 2.1); lack of knowledge may amplify the shock, uncertainty, and distress of the diagnostic process (see sub‐themes 2.2 and 2.3); and lack of knowledge may compound the already significant difficulties of the treatment experience (see sub‐theme 2.4).

Our observation that lack of knowledge may hinder symptom recognition is consistent with findings from both qualitative work with older but still relatively young adults (24‐35 years) diagnosed with cancer ^9^ and from survey‐based research with healthy AYA.^15^, ^16^, ^17^, ^18^ However, our work advances that literature by illuminating reasons for AYA’s limited knowledge, and highlighting potentially serious consequences, including delays in help‐seeking, and incomplete reporting of symptoms on presentation. The latter finding underscores the importance of professionals in primary and/or emergency care taking a complete and detailed history, and encouraging AYA to disclose any/all symptoms.

Less well documented previously is how AYA’s lack of clarity regarding the reasons for referrals and tests can have important and undesirable consequences, such as AYA attending pivotal appointments alone.^9^ Again, our work extends understanding of how such situations may arise. For example, our data suggest that professionals’ use of ambiguous and/or unfamiliar language may compound lack of knowledge and foster misunderstanding. Whilst professionals may, understandably, seek to avoid distressing AYA before a diagnosis is confirmed, lack of awareness of the diagnostic trajectory may also have serious consequences. Ensuring AYA (and indeed other patients) understand fully what is happening to them and why would seem fundamental to quality care.^38^, ^39^ Hence, where cancer is suspected, professionals might consider using less ambiguous language, checking understanding and advising AYA to bring a family member or friend to subsequent consultations.

Our finding that lack of knowledge may contribute to the difficulty of AYA’s treatment experience is quite new. Prior research suggests that AYA are not alone in lacking knowledge of contemporary cancer treatments. These are complex and varied, and similar assertions have been made about knowledge in other patient/research populations.^40^, ^41^, ^42^ However, the impact of lack of knowledge cannot be assumed to be the same for all patient/age groups. We believe this topic invites further exploration.

### 4.1 Implications for policy and practice

Our findings prompt important questions about whether, when and how, AYA’s knowledge of cancer might be enhanced, with a view to promoting timely diagnosis and optimizing experiences of treatment/care. Two broad approaches warrant consideration: universal education on AYA cancer, and/or health; and targeted education (enhanced information and counselling) at and after diagnosis.

Other researchers have previously argued for, the introduction and/or expansion of developmentally appropriate, school‐based education on cancer risks and symptoms.^16^ Evaluation of the effectiveness of such initiatives for increasing (younger) adolescents’ knowledge of cancer risk factors and symptoms has found evidence of some short‐term success.^43^ However, our findings suggest some barriers to lasting impact might be anticipated. Firstly, as our interviewees reported viewing cancer as a topic of limited personal relevance prior to diagnosis, the effectiveness of such educational initiatives may be contingent on changing this mindset. Moreover, the non‐specific nature of many of the symptoms reported by AYA (eg fatigue) may make consistent and enduring recognition hard to achieve. Other work, on delays in diagnosis of haematological malignancies in adults, has reported similar challenges and recommended that education should focus on promoting recognition of normal health (encouraging help‐seeking should that change).^44^ Such an approach might have salience for AYA cancers too. Furthermore, this sort of message might usefully be targeted at both AYA and the key adults in their lives (e.g. parents, caregivers, teachers, employers).

On diagnosis with cancer, our findings suggest that (for most AYA) the learning curve will be steep, requiring assimilation of both new language and concepts. AYA’s reflections on their experiences of diagnosis and initial/front‐line treatment point to the importance of education at and beyond diagnosis, but also to the challenges of delivering this. We have discussed elsewhere how AYA’s physical and emotional states can create difficulties processing and absorbing information when first diagnosed.^14^ Whilst there are clearly significant obstacles to enhancing knowledge at this time, there may be more scope to achieve this over subsequent weeks and/or months, once AYA have processed their diagnosis and acquired some experiential understanding of treatment. Most of the AYA who took part in our study were receiving care through specialist AYA cancer services and supported by committed professionals with some level of expertise in providing care to this (age) group. Nevertheless, their accounts suggest their educational/informational needs were not fully met. Other authors have reported that (adult) oncologists do not consistently ascertain their patients’ prior knowledge and argued that such a practice is essential if information is to be tailored to patients’ evolving needs.^45^ The AYA population too might benefit from clinicians establishing a practice of routinely assessing individual patients’ prior—and evolving—knowledge and informational needs. Consideration might also be given to the role that parents/caregivers can play in building AYA’s cancer knowledge, although it is important to note that parents/caregivers’ priorities and information needs may not always align with those of AYA.^14^

### 4.2 Strengths and limitations

The retrospective nature of our study might be considered both a strength and a limitation. Unlike previous survey research with healthy AYA, we collected data from AYA with cancer, who, in contrast to their (healthy) peers were in a position to identify ‘known unknowns’ (ie what, of importance, they had not previously known). In addition, the use of semi‐structured interviews allowed AYA the flexibility to raise, and describe, issues viewed by them as particularly salient—indeed it is as a result of this that ‘prior knowledge’ emerged as a significant analytical theme. However, recall difficulties may have limited the range of comments/responses, and constrained our understanding of how AYA’s perceptions of their knowledge, and need for this, may change over time. Prospective, longitudinal work, involving serial interviews, might provide additional and useful insights on ‘teachable moments’, that is when AYA with cancer are particularly desirous of, and receptive to, new information and knowledge.

## 5 CONCLUSION

This qualitative work illuminates how (lack of) prior knowledge of cancer may impact upon AYA’s experiences of diagnosis with cancer and initial/front‐line care. It suggests knowledge gaps may have serious consequences at various points in AYA’s pathways to and through treatment. These findings prompt questions as to whether, when and how, AYA’s knowledge of cancer and its treatment might be improved. Unfortunately, there are no easy answers, though we suggest two broad approaches warrant consideration. Ultimately individuals may vary in their keenness for, and receptivity to, cancer‐related information at different times^38^; assessment and repetition may be key to improving AYA’s knowledge of cancer and its treatment/care.

## CONFLICT OF INTEREST

We have no conflicts of interest to declare.

## Data Availability

Data (eg transcripts) have not been made not publicly available, due to the small number of AYA diagnosed with cancer in Scotland each year, and the associated risk of participants being identified notwithstanding the removal/redaction of obvious identifiers. Requests, to the corresponding author, for access to the data underpinning this paper will be considered and accommodated where reasonable.
